# Supplementary material for: Attentional enhancement predicts individual differences in visual working memory under go/no-go search conditions
Source: PLoS Biol. 2022 Nov 28;20(11):e3001917. doi: 10.1371/journal.pbio.3001917 (PMC9731499; doi:10.1371/journal.pbio.3001917)
Supplement: S1 Fig — The underlying data supporting this figure can be found at https://osf.io/4wdzq. (A) Distractor-suppression activity over the posterior scalp (PD) on No-Go trials of Experiment 1 did not predict WM capacity. (B) Response-suppression activity over the central scalp (no-go P3) on No-Go trials of Experiment 1 did not predict WM capacity. (PDF) [file pbio.3001917.s001.pdf]

Full title: Attentional enhancement predicts individual differences in visual working memory under go/no-go search conditions

Short title: Attentional enhancement predicts memory capacity

Daniel Tay*[1] and John J. McDonald[1]

Department of Psychology, Simon Fraser University, Burnaby, British Columbia, Canada

* daniel_tay@sfu.ca

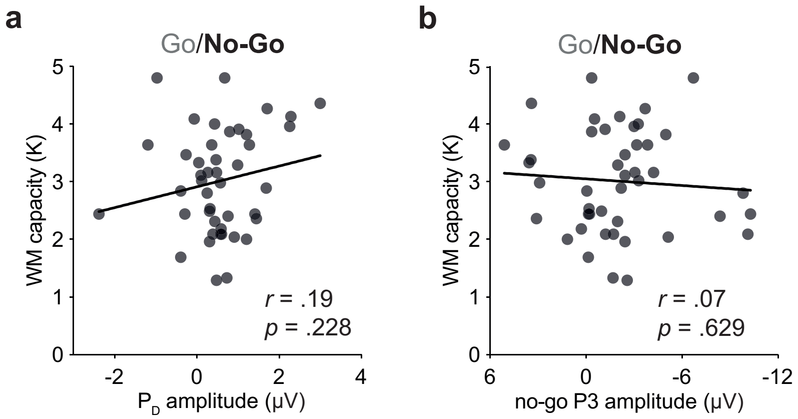

S1 Fig. Bivariate relations between individuals' WM capacities and amplitudes of isolated ERP indices of inhibition in Experiment 1. The underlying data supporting this figure can be found at https://osf.io/4wdzq. (**A**) Distractor-suppression activity over the posterior scalp (P_D) on No-Go trials of Experiment 1 did not predict WM capacity. (**B**) Response-suppression activity over the central scalp (no-go P3) on No-Go trials of Experiment 1 did not predict WM capacity.
