# Supplementary material for: Attentional enhancement predicts individual differences in visual working memory under go/no-go search conditions
Source: PLoS Biol. 2022 Nov 28;20(11):e3001917. doi: 10.1371/journal.pbio.3001917 (PMC9731499; doi:10.1371/journal.pbio.3001917)
Supplement: Attachment — Submitted filename: gd4c response to reviewers.pdf [file pbio.3001917.s003.pdf]

Reviewer 1:

Synopsis:
Tay & McDonald report two experiments designed to test whether excitatory control processes correlate with working memory (WM) capacity, which would challenge prevailing views of WM gating based on inhibitory control. The authors track a multitude of ERP components linked with excitatory and inhibitory attentional control in previous studies while participants perform a go/no-go variant of a pop-out search task (Exp 1) or a classic all-go pop-out search task (Exp 2). Since the former task requires a greater degree of control than the latter (i.e., go/no-go plus target detection vs. just target detection), a link between WM capacity and ERP correlates of excitatory control processes should be most evident there. This is borne out in the experiments; two excitatory ERP components predict WM capacity in Experiment 1, but not Experiment 2. Thus, excitatory control processes also play a role in gating access to WM.

Evaluation:
This is a technically sound study that makes an important point about mechanisms that control access to WM. The conclusions hinge on whether the ERP components are linked to excitatory and inhibitory control processes in the manner the authors assume. I'm not fully up-to-date with the ERP literature, but each claim made by the authors is supported by at least one prior published study and I'm prepared to take the authors' interpretations on faith (other referees can perhaps speak to any shortcomings here).

Author Response: We thank Reviewer 1 for the positive assessment!

My only concern is that the authors might be underselling their conclusions: prevailing models of WM input gating focus exclusively (to my knowledge) on the operation of inhibitory mechanisms that "filter out" task-irrelevant information; the current data argue against this view. This is only very briefly discussed in the last paragraph of the paper. If space allows, I encourage the authors to expound on their suggestion that "Current attentional-control perspectives of WM need to be updated to account for these findings". For example, which perspectives are the authors referring to, and how - in the authors' view given the data - should they be modified?

Author Response: After considering the comments from all reviewers, we decided to change our inhibition-vs-excitation focus to one that focuses more specifically on the distinction between attentional selection processes that enhance relevant items and ones that suppress irrelevant items. We still discuss 'inhibition' in the results (e.g., of response processes; no-go P3), and we discuss implications of our results for more general theories of WM, including the inhibition view.

Reviewer 2 (Sirawaj Itthipuripat):

In the present paper, Tay and McDonald examined the relationship between the excitatory attentional control processes and individual differences in working memory (WM) capacity. They argued that although both excitatory and inhibitory processes have been shown to underly attentional control, only the inhibitory processes have been associated with individual differences in WM capacity. In their version of the go-nogo visual search task, they found that the amplitudes of the ERPs that track the excitatory processes in the go trials, i.e., the SDP and the N2pc components (but not the P2a component), were positively correlated with intersubjective variability in WM capacity. In contrast, the inhibitory ERPs including the Pd and the P3 components in the nogo trials did not predict WM capacity across subjects. In a control study, where subjects searched for a pop-out target in every trial, they found no correlation between the SDP and N2pc amplitudes and WM capacity. Taken together, they proposed that the excitatory attentional processes control access for visual WM. Overall, I think the study addressed a novel and important question that has a potential to shape theories that explain the interaction between attention and WM mechanisms. The experiments were well designed and executed with appropriate ERP and statistical analyses. The results provide new mechanistic insights into how different types of neural computations underling attentional selection may interact with WM. I only have some minor concerns.

Author Response: We thank Dr. Itthipuripat for the positive assessment!

1) Even though the question is current and interesting, I think the paper needs a better motivation—not just that past studies only showed evidence for inhibitory processes and now we simply want to see if excitatory processes are involved. There should be a clearer hypothesis-driven motivation e.g., why excitatory attentional control processes are important for WM, how their involvement might be functionally distinct from the inhibitory processes, and at what context one mechanism might me more dominant than the other.

Author Response: We now focus on the distinction between attentional-selection processes that enhance relevant items and ones that suppress irrelevant items. We added to the motivation of our hypotheses and methods in the Introduction. Here we note that we think the research question would be of interest for the broad readership of PLoS Biology. Previously, researchers have investigated whether distractor suppression is predictive of WM capacity, and the seminal studies on that related topic have been published in excellent journals including *PNAS* (Gaspar et al., 2016) and *Cerebral Cortex* (Feldmann-Wüstefeld & Vogel, 2019).

2) Related to (1), why in some contexts (like in past studies) inhibitory mechanisms dominate, and in some contexts (like in the present study) excitatory processes dominate. This should be discussed further. What special about the nogo component of the task that made the results diverted from past studies that observed the more dominant role in the inhibitory processes?

Author Response: We have addressed this question in our new 'Summary and Conclusion' section.

3) Why the P2a component, which is also excitatory in the authors' view, did not predict WM capacity like the N2pc and SDP did. This should be discussed in detail.

Author Response: We have moved away from the more general "inhibition-vs-excitation" question and towards one that focuses on attention control processes associated with target enhancement (vs. distractor suppression). Here, we note that the P2a does not reflect the same kind of attention-control processes that would help to transfer information about one relevant item into working memory. Instead, it highlights an entire display as one that is in need of further inspection. In our revised manuscript, we argue that attention-control processes predict vWM capacity only when control over the attention process is actually required. Apropos of this, we believe that the relevancy processing is automated after a few trials so that it requires very little control.

4) I think the reader would benefit from seeing the topographic maps of different ERP components.

Author Response: Thanks for the suggestion. Topo maps have been added to Figure 2.

5) I would love to see additional figures showing correlation results between Pd/P3 and WM capacity to ensure that null results were not driven by some outliers. Right now, there were only correlation figures for the P2a, N2pc and SDP data.

Author Response: These have also been added to our new Supplemental Figure 1.

6) When introducing excitatory and inhibitory mechanisms for the first time (in the abstract), I think the authors should be more specific by relating those mechanisms to the attentional processes. Without

no background, the reader might get confused that the authors meant excitatory and inhibitory mechanisms that directly underly WM rather than attention itself.

Author Response: Thanks for this suggestion. We have gone a bit further (also in response to Reviewer 3) to focus on attention-control mechanisms associated with target enhancement and distractor suppression (rather than 'excitation' and 'inhibition', respectively).

7) Can the authors discuss limitations of using ERPs to track excitatory and inhibitory processes? Since ERPs are the population-level neural responses, how could the authors be sure that certain ERP components are truly excitatory or inhibitory? It would be good to cite non-human primate work that provides the links between the single unit activity and population-level activity as well as the associated excitatory and inhibitory processes.

Author Response: We don't think that such a discussion is required now that we have shifted focus away from excitation and inhibition. The shift in focus was done in part to prevent readers to think that we are necessarily talking about inhibition and excitation at the cellular level.

Reviewer 3:
In two experiments, subjects first performed colored-blocks-array 'change detection,' to estimate working memory (WM) capacity, then a pop-out search task while the EEG was recorded concurrently. In Exp 1, trials varied unpredictably between conventional trials ("Go") and "No-Go" trials during which they were to withhold their response. Exp 2 featured only Go trials. The authors focus on three 'excitatory' components (P2a; SDP; and the N2pc) and two 'inhibitory' components (PD; and no-go P3). The motivation for this study is that although there is a great deal of evidence linking inhibitory processes to individual differences in WM capacity, the same is not true for "excitatory" processes. The authors "surmised [that] a link between excitatory attention control and WM capacity would be revealed in a task that requires more control than the conventional pop-out search paradigm," and the Go/No-Go procedure from Exp 1 was intended to engage this control.

The results are generally consistent with the authors' surmise, although I'm not sure that they are as impactful as they are made out to be. Part of the problem I'm having may come from a disconnect between the rather "high" theoretical level that is engaged to motivate this work versus the very concrete, mechanistic dependent measures that are collected and interpreted. That is, the theoretical framing is at a relatively high level that treats the constructs of "inhibition" and "excitation" as latent variables, and invokes papers like Redick et al. (2011), Hasher et al. (1999), and Kane et al. (2001) that conceptualize "inhibition" as a trait*. When engaging at this level, it can make sense to make a statement like "the gateway to WM is predominantly inhibitory in nature." However, these experiments don't engage directly with this "higher" theoretical level. Rather they engage a mechanistic/implementational level of specific processes, the processes associated with the 5 ERP components summarized above. At this more mechanistic level, a statement like "the gateway to WM is predominantly inhibitory in nature" simply doesn't make sense, because it has to be the case that the encoding of information into WM requires some operation akin to selection or input gating. A much more theoretically coherent way to motivate this paper would be with a sentence from the final paragraph of the main text (the "Discussion" paragraph): "… prior studies reported that WM does not vary with amplitude of the target-elicited N2pc." It's perhaps less grandiose, but it's also a more accurate reflection of what's at stake in this paper. (Doing this would also require tempering the final sentence about these needing to update current attentional-control perspectives of WM.)

Author Response: We shifted focus away from the general excitatory-vs-inhibitory issue, although we do still try to connect with theories of visual WM that make reference to these terms. The revised manuscript asks whether the attention-control processes that enhance visual-search targets (as opposed to those that suppress distractors) are predictive of visual working memory capacity. This issue is of interest to many researchers in the field and, in our opinion, would be of interest to the broad readership of PLoS Biology. As highlighted in numerous papers (published in top-tier journals; e.g., Feldmann-Wüstefeld &

Vogel, 2019, *Cereb. Cortex*; Gaspar et al., 2016, *PNAS*; McNab & Klingberg, 2008, *Nat Neurosci*; see also LaBerge's 1995 book), attentional selection can be achieved by enhancing information arising from relevant objects or by suppressing information arising from irrelevant objects. By this perspective, we believe that "the gateway to WM is predominantly inhibitory by nature" makes good sense. It is a statement that links a high-level construct to the nature of processes at the mechanistic level, in that the selection/input gating mechanisms rely primarily on suppression of irrelevant input rather than enhancement of relevant input. The topic of distractor suppression is especially hot at the moment, and so we feel that our results (showing a situation where target enhancement, not distractor suppression, is particularly important) are timely and novel.

I do think that it is useful, as a demonstration of specificity, to show that the "inhibitory" ERPs (the PD** and the no-go P3) do not show the same dependence on block homogeneity as do the "excitatory" ones. It's conceptually awkward, however, because elsewhere it's stated that "the link between WM capacity and inhibitory attention control has been well established." (It is also puzzling, given this, why it's stated that "Our second objective was to determine whether the inhibitory-control activity … would also predict WM capacity.") Should one infer from these null results that these two components are not good indices of inhibitory attention control? Alternatively, I think one could argue that there's no reason to expect strong effects with this design, because subjects have no reason to actively suppress individual items (i.e., no reason prevent their selection) because no-go trials only require withholding a response.

Author Response: We made revisions to clarify our position and to reduce the likelihood that readers will think our results or conclusions are conceptually awkward. Essentially, we believe that different tasks require, or at least promote, the use of different selection mechanisms (and in this task, other inhibitory processes to withhold responses on No-Go trials). We agree with Reviewer 3 that there was no reason to expect strong links between suppression/inhibition in this design, particularly because the P_D occurred relatively late on No-Go trials and thus could not have reflected suppressive processes that prevented covert orienting to the distractor (Tay et al., 2022, JEPHPP). This explanation has made it into our Discussion.

The final thing I'll note is that the assertion that "excitatory" ERP effects haven't previously been associated with WM capacity feels somewhat disingenuous in that it overlooks the huge (and hugely influential) literature on the CDA. Indeed, I think that one way to make these results about more than 'just' the empirical question of whether one can or cannot obtain an association between the SDP and the N2pc and WM capacity would be to speculate about how the positive findings from this study fit into the literature on the CDA.

Author Response: Our initial submission was written as a (very) short report or "letter". The revision includes a considerably longer Introduction section, wherein we discuss the CDA. Also, we have tried to do a better job at focusing on attention-control processes that enhance relevant objects instead of any and all excitatory ERP components. To this end, we have replaced "excitatory ERPs" with terms that link the components of interest to more specific (hypothetical) processes, including enhancement processes associated with target detection and selection (SDP and N2pc, respectively).

General note: The transition from Introductory Paragraph to the Main Text is very abrupt. This might be a constraint of the short-report format, but a few sentences of introduction delving into the design might improve overall readability.

Author Response: As noted in our previous response, the original submission was formatted as a 'letter' type manuscript. The 'introductory paragraph' was turned into an Abstract, and four Introductory paragraphs have been added.

Methodological question: It doesn't make sense to me that one would include the capacity estimated at set-size 2 in the values that are averaged to estimate an individual's overall capacity. The theoretical upper limit at SS2 is 2, so its inclusion would necessarily underestimate the k of a subject whose true k is > 2.

Author Response: We recomputed the K values with data from set-size 2 trials excluded. The associated methods, stats and figures have all been revised accordingly.

* Note, however, that if one is reasoning from this higher level there are other perspectives that should also be taken into account, such as Unsworth, N., Fukuda, K., Awh, E., & Vogel, E.K. (2014). Working memory and fluid intelligence: Capacity, attention control, and secondary memory. Cognitive Psychology, 71, 1-26.

Author Response: Because our focus is not to establish links between working memory and other high-level factors, we believe that it would not make sense to discuss these other perspectives. Our focus is on establishing a previously unknown link between target-enhancement processes and working memory capacity.

**why is it referred to as just "PD" in some places and as "late PD" in others?

Author Response: We now use the term P_D consistently throughout the manuscript but note that it occurs relatively late in this paradigm.

Academic Editor:

Regarding the overall impact: Considering that the relationship between excitatory measures of attention & wm capacity is only observed in the go/no go procedure, couldn't the continual need to inhibit attention between trials (disengage from some; engage for others) be interpreted that the connection to wm capacity is still dependent on some form of inhibition? The authors briefly mention this possibility, but dismiss it because there were few "no-go" errors. I don't this is a sufficient argument because their neural measures of attention are so much earlier than behavioral responding. I'd like to see them grapple with this issue a bit more explicitly.

Author Response: Thank you for challenging us to grapple with this issue. We made some points in our expanded Introduction as to why we introduced the Go/No-Go decision to this task. In the Discussion section, we specifically address the 'need-to-inhibit' issue directly. We do not empirically rule out the need for inhibition to generate the relationship between target N2pc and vWM capacity, but we offer an argument as to why this possibility is unlikely along with our opinion as to what we think is important. In a nutshell, here is our argument: (1) low-capacity individuals have problems suppressing/inhibiting in some tasks; (2) it is possible that they had some difficulty suppressing/inhibiting on No-Go trials of this task; (3) if so, then their ability to attend to the target on subsequent Go trials should be *disinhibited* relative to the ability of high-capacity individuals; (4) such disinhibition should lead to a *larger* N2pc on Go trials (for low-capacity individuals, relative to high-capacity individuals). The opposite pattern was observed, thus showing that there was no 'disinhibition' for low-capacity individuals. If inhibition problems do not somehow lead to the N2pc-capacity correlation, what did? We believe that the Go/No-Go decision prevented participants from being able to automate the target-based search process, resulting in the need for more controlled attention processes. The timing of the P2a (being earlier than the N2pc) is suggestive that the Go/No-Go decision was made first, and thus we believe that participants automated the discrimination of display-wide array colour and then had to *switch* into search mode. In future studies, we will test this explanation by replacing the Go/No-Go requirement with a task-switch requirement. We predict that task switching between search trials and some other (non-search) trials will also lead to a N2pc-capacity correlation.

Technically: A key part of the authors' argument relies on their ability to interpret a null correlation in Experiment 2. This can be problematic for a number of reasons, particularly if one or more of the measurements has low reliability. That is, the strength of a correlation between two variables cannot be higher than the reliability level of the either of the measures. The authors need to calculate and report the reliability of each of their measures to show that the lack of correlation in Exp 2 wasn't simply because they had poor reliability for their measurements.

Author Response: We now report split-half reliabilities for the SDP and N2pc in Experiment 2. The split-half reliabilities were much higher than the observed null correlations.
