# Supplementary material for: Attentional enhancement predicts individual differences in visual working memory under go/no-go search conditions
Source: PLoS Biol. 2022 Nov 28;20(11):e3001917. doi: 10.1371/journal.pbio.3001917 (PMC9731499; doi:10.1371/journal.pbio.3001917)
Supplement: Attachment — Submitted filename: excitation_wm.docx [file pbio.3001917.s002.docx]

In the present paper, Tay and McDonald examined the relationship between the excitatory attentional control processes and individual differences in working memory (WM) capacity. They argued that although both excitatory and inhibitory processes have been shown to underly attentional control, only the inhibitory processes have been associated with individual differences in WM capacity. In their version of the go-nogo visual search task, they found that the amplitudes of the ERPs that track the excitatory processes in the go trials, i.e., the SDP and the N2pc components (but not the P2a component), were positively correlated with intersubjective variability in WM capacity. In contrast, the inhibitory ERPs including the Pd and the P3 components in the nogo trials did not predict WM capacity across subjects. In a control study, where subjects searched for a pop-out target in every trial, they found no correlation between the SDP and N2pc amplitudes and WM capacity. Taken together, they proposed that the excitatory attentional processes control access for visual WM. Overall, I think the study addressed a novel and important question that has a potential to shape theories that explain the interaction between attention and WM mechanisms. The experiments were well designed and executed with appropriate ERP and statistical analyses. The results provide new mechanistic insights into how different types of neural computations underling attentional selection may interact with WM. I only have some minor concerns.

1) Even though the question is current and interesting, I think the paper needs a better motivation—not just that past studies only showed evidence for inhibitory processes and now we simply want to see if excitatory processes are involved. There should be a clearer hypothesis-driven motivation e.g., why excitatory attentional control processes are important for WM, how their involvement might be functionally distinct from the inhibitory processes, and at what context one mechanism might me more dominant than the other.
2) Related to (1), why in some contexts (like in past studies) inhibitory mechanisms dominate, and in some contexts (like in the present study) excitatory processes dominate. This should be discussed further. What special about the nogo component of the task that made the results diverted from past studies that observed the more dominant role in the inhibitory processes?
3) Why the P2a component, which is also excitatory in the authors’ view, did not predict WM capacity like the N2pc and SDP did. This should be discussed in detail.
4) I think the reader would benefit from seeing the topographic maps of different ERP components.
5) I would love to see additional figures showing correlation results between Pd/P3 and WM capacity to ensure that null results were not driven by some outliers. Right now, there were only correlation figures for the P2a, N2pc and SDP data.
6) When introducing excitatory and inhibitory mechanisms for the first time (in the abstract), I think the authors should be more specific by relating those mechanisms to the attentional processes. Without no background, the reader might get confused that the authors meant excitatory and inhibitory mechanisms that directly underly WM rather than attention itself.
7) Can the authors discuss limitations of using ERPs to track excitatory and inhibitory processes? Since ERPs are the population-level neural responses, how could the authors be sure that certain ERP components are truly excitatory or inhibitory? It would be good to cite non-human primate work that provides the links between the single unit activity and population-level activity as well as the associated excitatory and inhibitory processes.

Signed Sirawaj Itthipuripat
